# Kidney Disease in Elderly: Importance of Collaboration between Geriatrics and Nephrology

**DOI:** 10.14336/AD.2018.0223

**Published:** 2018-08-01

**Authors:** Faheemuddin Azher Ahmed

**Affiliations:** OSF Saint Anthony Medical Center, Rockford, Illinois, USA

**Keywords:** nephrology, geriatrics, chronic kidney disease, dialysis

## Abstract

The population in the United States is aging and presents many challenges in the healthcare world. According to the report released by United States Census Bureau in June 2017, there are around 50 million residents aged 65 years and over as of 2016. Among the multiple healthcare challenges, kidney disease is a significant one because of its high burden, high cost and low awareness. Medicare spending on chronic kidney disease for 65 plus aged patients exceeded $ 50 billion in 2013. Different studies based on different calculations have estimated that at least one-third of chronic kidney disease patients are aged above 65 years. Most of the chronic kidney disease patients have multiple medical co-morbidities but geriatric syndromes are added factors that may be challenging for nephrologists. There is scarcity of well-trained geriatricians and in most instances, nephrologists take over the role of internist or geriatrician. This article outlines the need and importance of collaboration and coordination between geriatrics and nephrology for the best patient care and better healthcare outcomes.

The elderly population continues to grow and according to Population Reference Bureau bulletin on Aging in the United States, the projected number of people aged over 65 years will be around 98 million by 2060. Longevity is mirrored with an increase in the occurrence of acute clinical conditions and prevalence of chronic conditions. Advances in medical science and technology has increased the treatment options but at a significantly higher cost. Therefore, there is an increased utilization of healthcare services and resources in this age cohort. Among all the medical co-morbidities, kidney disease and its associated complications are medically and fiscally challenging. Medicare spending on chronic kidney disease for 65 plus aged patients exceeded $ 50 billion in 2013 (1). Therefore, clinical nephrologists will see an increased number of encounters with the older patient population. Nephrology training doesn’t have geriatric medicine training incorporated and it will be challenging for nephrologists to address geriatric medical issues. It is important to establish co-management team for optimization of patient care and better healthcare outcomes.

## Geriatrics in Nephrology

Geriatric syndromes are used to describe clinical health conditions that are unique to the elderly population which cannot be categorized as disease/system specific. Some examples of the geriatric syndromes are cognitive impairment, falls, dizziness, incontinence, frailty, etc. The impact of renal disease and dialysis on geriatric syndromes is often associated with poor outcomes.

Cognitive impairment and depression are common in elderly chronic kidney disease patients. Dialysis has shown to worsen cognitive function which in turn worsens quality of life, increases hospitalizations and poor healthcare outcomes (4).

As per Centers for Disease Control and Prevention, National Center for Injury Prevention and Control; Falls are the leading cause of injuries in the people aged 65 or older. Dialysis is associated with increased risk for falls because of dialysis-related hypotension, cramping, exhaustion. Patients with chronic kidney disease are on multiple medications and have other medical co-morbidities which contribute to multifactorial contribution for fall.

Elderly patients are prone to develop malnutrition and weight loss. Chronic Kidney Disease and End-Stage Renal Disease patients’ risk is further higher with anorexia secondary to uremic toxins, nutritional losses during dialysis, depression, etc. This further contributes to increased morbidity and mortality (5).

Physical and quality of life scores among dialysis patients were found to be lower than the controls (6).

## Nephrology in Geriatrics

Renal disease in elderly patients may present challenges not only to an internist but also to a geriatric trained physician because of multiple reasons.

Different studies based on different calculations have estimated that at least one-third of chronic kidney disease patients are aged above 65 years (2,3). It is important to distinguish age-related decline in kidney function from the intrinsic renal disease in this age group. The progression of kidney disease may not occur in a predictable fashion. Measurement formulae for kidney disease categorization in this age group have limitations. There are insufficient guidelines in management of the diabetic chronic kidney disease patients in the geriatric population.

Drug therapy in elderly patients is challenging. This gets furthermore complicated in presence of renal disease. Around one-third of prescription drugs are used by geriatric patients. There are limited clinical data on dosing of drugs in elderly kidney disease patients. There is three to tenfold increase in the incidence of adverse events in elderly patients with kidney disease in comparison to those without kidney disease (7). Therefore, an internist and/or geriatrician may not be well versed with drug dosing, side-effects and complications of medications in this age group.

The management of fluid and electrolytes may need a different approach in this population in comparison to adult population.

## Geriatric Medicine Curriculum in Nephrology Fellowship Training

There is no well-defined curriculum to teach geriatric medicine in nephrology fellowship training. This will leave nephrologists going into practice with challenging encounters because of lack of knowledge of unique geriatric issues. American Society of Nephrology has developed an online curriculum for practicing nephrologists to learn about geriatric nephrology issues.

## Shortage in Geriatric Medicine Trained Physicians

According to data compiled by American Geriatrics Society’s Geriatrics Workforce Policy Studies Center (GWPS), there will be a need of 30,000 geriatricians by 2030. There are around 7300 certified geriatricians currently in practice which shows the shortage in this field as against the continuously growing population.

## Need and Importance of Collaboration/Coordination Between Geriatrics and Nephrology

Keeping in view the lack of geriatric curriculum in nephrology training and the shortage of geriatricians, it mandates the need for collaboration and care coordination between geriatricians and nephrologists.

The establishment of the co-management team should be started early in the disease course for tailoring patient’s care to their goals. The geriatrician can perform a comprehensive geriatric assessment. Cognitive, functional, and psychosocial assessment can help delineate potential issues and appropriate treatment as the disease progresses. Nephrologists can help with regards to fluid and electrolyte management, anemia, bone mineral disease treatment, drug monitoring/dosing right from early stages of the disease process.

Progression of kidney disease often leads to the complex dialysis decision. Dialysis is a lifesaving treatment but carries its own side-effects and complications. The risk profile of dialysis in geriatric population is worse than adults because of age-related physiological changes, more medical comorbidities and geriatric syndromes. According to USRDS 2016 report, dialysis patients over the age of 75 years experienced around four times higher mortality rates compared to the general Medicare population (1). One-year mortality was 46% after the start of dialysis for octogenarians and nonagenarians between 1996 and 2003 (8). The decision-making process should involve multiple perspectives: patient, caregiver/family, treatment in conjunction with the geriatrician-nephrologist collaborative team.

## Conclusion

Geriatric nephrology is a complex field and requires coordinated and comprehensive care approach through collaboration between geriatricians and nephrologists for optimal patient care. A geriatrician can help nephrologist by providing comprehensive geriatric assessment, long-term care planning, palliative care discussions. Nephrologists can help in addressing and managing medical conditions, dialysis preparation and delivery. This can help in effective treatment strategies and help in reduction of healthcare costs.
